# Physical and Gastrointestinal Digestive Properties of Sodium Caseinate Emulsions Regulated by Four Different Polysaccharides

**DOI:** 10.3390/gels11120968

**Published:** 2025-12-01

**Authors:** Mengyao Kang, Denglin Luo, Lihua Zhang, Jiaxiang Zang, Lala Li, Wei Xu

**Affiliations:** 1 College of Food and Bioengineering, Henan University of Science and Technology, Luoyang 471023, China; kangyao200001@163.com; 2 College of Life Science, Xinyang Normal University, Xinyang 464000, China; zhanglihua@xynu.edu.cn (L.Z.); 15713711826@163.com (J.Z.); 15517984382@163.com (L.L.)

**Keywords:** emulsion, sodium caseinate, polysaccharide, stability

## Abstract

Polysaccharide intervention is an effective strategy to regulate properties of emulsions. In this study, xanthan gum (XG), konjac glucomannan (KGM), guar gum (GG), and inulin (IN) were selected to regulate physical and gastrointestinal digestive properties of sodium caseinate (CAS) oil-in-water (O/W) emulsions. The results indicate that IN could not improve CAS emulsion properties, while XG, KGM, and GG significantly reduced droplet size and improved emulsions’ stability. With the increase of the polysaccharide concentration, the G′ and G″ of the emulsions increased and the emulsions showed an obvious “solid-like” state, which effectively slowed down the “strain-thinning” phenomenon. The microstructure demonstrated that the polysaccharide chains are effectively connected with the surface membrane of droplets, which effectively improves interfacial membrane strength and inhibits droplet aggregation. In vitro digestion simulations proved that polysaccharides effectively modulate emulsion lipid release, providing an excellent lipid environment for curcumin absorption in the gastrointestinal tract. The order of the four polysaccharides in improving CAS emulsions was XG > KGM > GG > IN. This study dissects the differential regulation of physical and gastrointestinal digestive properties of emulsion by polysaccharides, providing theoretical support for functional emulsions for diverse requirements.

## 1. Introduction

Emulsions, as colloidal dispersion systems with highly complex structures and diverse functionalities, occupy an important position in industries such as food and medicine [1]. However, water–oil phase incompatibility essentially constitutes a key obstacle to the destabilization of emulsion systems, especially when loaded with bioactive ingredients. External environmental factors (temperature fluctuations, pH changes, and mechanical forces) tend to induce instabilities of emulsions, affecting their organoleptic properties and physical stability, such as aggregation of oil droplets, precipitation, Ostwald ripening, and flocculation [2]. To further complicate matters, in the highly complex physiological environment of the gastrointestinal tract, the multiple effects of gastric acid, digestive enzymes, and ionic strength further disrupt the structure of the emulsions, interfering with the release and absorption of the active ingredients [3,4]. In view of this, optimizing the stability of emulsion systems is an urgent technical challenge in food emulsion.

CAS is widely used as an emulsifier in food emulsification systems due to its amphiphilic nature and spontaneous adsorption at the air–water interface. CAS can form stable micellar structures in the aqueous phase, and these micelles effectively adsorb on the surface of oil droplets to form a protective film that prevents aggregation and fusion of oil droplets [5]. Additionally, CAS enhances the cellular uptake of hydrophobic nutrients by interacting with intestinal epithelial cells [6], making it a promising candidate for stabilizing emulsions and active substance delivery systems. However, emulsions stabilized solely by CAS are prone to instability due to heterogeneous interactions between protein molecules, leading to aggregation, depolymerization, or dehydration, which can cause emulsion rupture [7,8]. This limitation restricts the use of CAS in active ingredient delivery, prompting the exploration of protein–polysaccharide complexes as a potential solution for improving emulsion stability.

Due to their excellent emulsifying, thickening, and biocompatibility properties, polysaccharides are effective stabilizers for enhancing emulsion stability. It has been demonstrated that it can form a protective barrier to prevent droplet agglomeration and phase separation, which reduces interfacial tension and enhances the stability of the emulsions [9,10,11]. Xu et al. showed that IN and KGM formed a rigid gel structure around CAS-covered oil droplets and increased the viscosity of the continuous phase, leading to a decrease in droplet mobility and thus preventing droplet aggregation [12]. Ke et al. experimentally demonstrated that pectin of chayote (*Sechium edule*) could be electrostatically adsorbed onto CAS and formed a thick interfacial layer, which prevented the droplets from flocculation and emulsification, and improved emulsion stability [13]. Although previous studies have shown that protein–polysaccharide complex systems have significant advantages in emulsion stability, the differential regulation with different polysaccharides is still not completely clear.

Therefore, four polysaccharides with different properties and functionalities, XG, KGM, GG, and IN, were selected in this paper to investigate the effect of different polysaccharides on the properties of emulsions through rheological and stability analyses, microstructural characterization, and lipid digestion experiments. The results will contribute to a deeper understanding of the differential modulation of emulsion properties by polysaccharides.

## 2. Results and Discussion

### 2.1. Particle Size of Polysaccharide/CAS Emulsions

**Figure 1:**
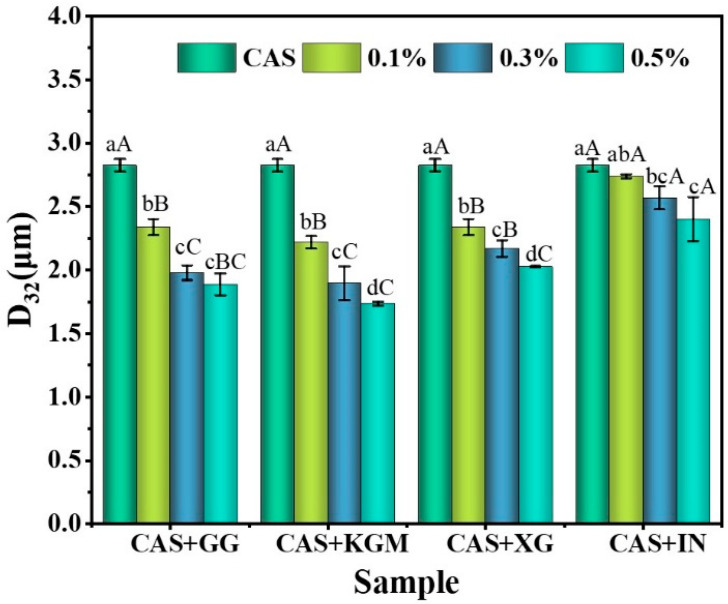
D_32_ of polysaccharide/CAS emulsions. Different lower/uppercase letters in the graphs indicate significant differences in emulsion D_32_ for different concentrations of the same polysaccharide or for different polysaccharides at the same concentration (*p* < 0.05).

Figure 1 illustrates the impact of various polysaccharides at different concentrations on the D_3,2_ of emulsions. The findings indicate that CAS emulsions exhibit the largest droplet size, with a significant reduction in droplet dimensions upon the addition of polysaccharides (*p* < 0.05). This suggests that polysaccharides enhance the interfacial stability of emulsions through specific molecular mechanisms, thereby inhibiting oil droplet aggregation and creaming. KGM/CAS emulsions showed the smallest particle size at all concentrations. This is primarily attributed to the high molecular weight and long linear chain structure, which provide additional contact sites on the droplet surface, facilitating interaction with CAS molecules to form a dense interfacial adsorption layer [14]. A stable three-dimensional network is constructed in the aqueous phase through intermolecular entanglement, which restricts the aggregation and migration of droplets and minimizes the particle size. The particle size of XG/CAS emulsions is slightly larger than that of KGM/CAS emulsions. This difference mainly stems from the rigid molecular chain structure of XG [15], whose extended arrangement interacts with protein molecules to form a thicker interfacial layer. Meanwhile, the negatively charged carboxyl groups create an electrostatic barrier that inhibits droplet aggregation. However, the strong electrostatic repulsion and spatial resistance limit further particle size reduction [16]. The particle size of the GG/CAS emulsion was intermediate between KGM and XG. Its flexible chains and branched structure facilitate the formation of a relatively dense interfacial layer on the droplet surface. Nevertheless, the uneven branch distribution reduces the uniformity and compactness of the interfacial layer, limiting its ability to regulate particle size. In contrast, IN exhibited the weakest effect, with no significant change in particle size at different concentrations, indicating that inulin was ineffective at enhancing the properties of CAS emulsions.

### 2.2. Storage Stability of Polysaccharide/CAS Emulsions

**Figure 2:**
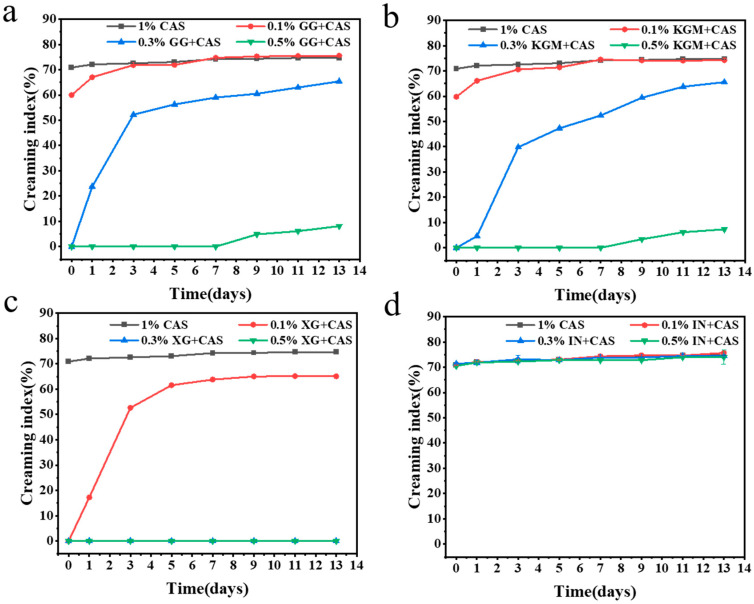
Creaming index of GG/CAS emulsions (**a**), KGM/CAS emulsions (**b**), XG/CAS emulsions (**c**), and IN/CAS emulsions (**d**).

**Figure 3:**
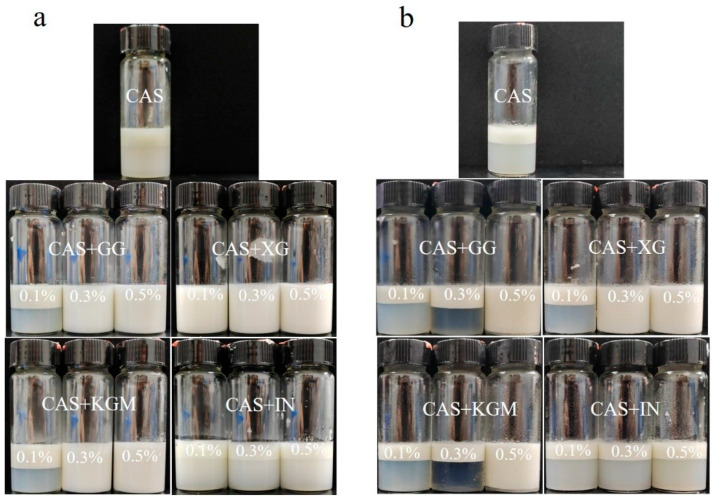
Visual appearance images of polysaccharide/CAS emulsions at 0 days (**a**) and 13 days (**b**).

Figure 2 characterizes the dynamic effect of different polysaccharides at different concentrations on emulsion layering using the creaming index. The results showed that CAS emulsions found it difficult to prevent droplet aggregation and gravitational uplift due to the weak interfacial film strength. The emulsions rapidly separated into cream and clear layers within minutes of preparation, showing very poor stability (Figure 3). The introduction of polysaccharides improved the layering behavior of the emulsions with a concentration-dependent effect. At low concentrations (0.1%), none of the polysaccharides (KGM, GG, and IN) significantly enhanced emulsion stability. The polysaccharide molecules were not sufficient to fully saturate the droplet surface, which triggered a bridging flocculation effect, and the delamination of emulsions was exacerbated [17]. Interestingly, XG effectively inhibits droplet agglomeration and migration through its electrostatic barrier effect and favorable rheological properties in the aqueous phase, maintaining relatively good stability at low concentrations. With the increase of the polysaccharide concentration, polysaccharides significantly reduced the emulsion creaming index, but the improvement varied according to the polysaccharide type. The emulsion improvement effects of KGM and GG were similar. KGM and GG reduced the creaming index by increasing the viscosity of the aqueous phase and forming a certain interfacial film on the droplet surface to prevent droplet aggregation. However, the stabilization mechanisms are slightly different. KGM relies on its high molecular weight and linear molecular structure to form a three-dimensional network structure in the aqueous phase to restrict droplet migration [18]. Whereas the molecular chain of GG has a high degree of flexibility, resulting in a weak network structure, the branched structure of GG can provide a rheological enhancement similar to that of KGM at high concentrations [19]. In contrast, XG/CAS emulsions showed optimal stability at high concentrations, almost completely preventing emulsion delamination. The creaming index of IN at all concentrations was like that of CAS emulsions, and the lack of improving ability of IN for CAS emulsions further validated the particle size results.

### 2.3. Steady-State Behavior of Polysaccharide/CAS Emulsions

**Figure 4:**
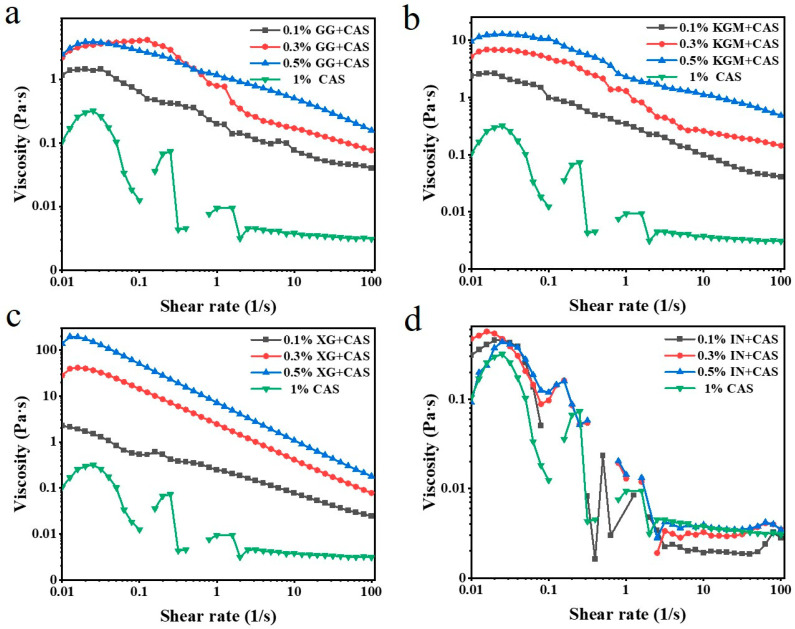
Steady-state behavior of GG/CAS emulsions (**a**), KGM/CAS emulsions (**b**), XG/CAS emulsions (**c**), and IN/CAS emulsions (**d**).

The steady-state behavior reflects the effect of polysaccharides on emulsion properties under dynamic processing environments. As shown in Figure 4, the apparent viscosities of all emulsions decreased with the increasing shear rate, exhibiting an obvious shear-thinning behavior. The molecular chains undergo an orientational alignment high shear rate, resulting in weakening of the molecular chain entanglement and interaction forces, and gradual disintegration of the three-dimensional network structure [20]. In addition, the relative motion between droplets intensified under the flow field, which further weakened the overall viscoelasticity of the emulsion system, leading to a significant decrease in viscosity with the shear rate. A comparison of polysaccharide types shows that XG-enhanced emulsions exhibited the highest initial viscosity at low shear rates, indicating that its electrostatic barrier effect strengthens intermolecular repulsion and spatial site resistance, creating a stable fluid resistance system. In comparison, KGM-stabilized emulsions showed lower stability than XG, and the uneven branched structure of GG further decreased emulsion viscosity. According to Stokes’ law, the high-viscosity emulsion system can effectively slow down the speed of droplet movement and inhibit droplet aggregation [21]. This mechanism suggests that XG has a better improving effect on emulsion stability through high viscosity, with the effect of IN being almost negligible.

### 2.4. Frequency Sweeps of Polysaccharide/CAS Emulsions

**Figure 5:**
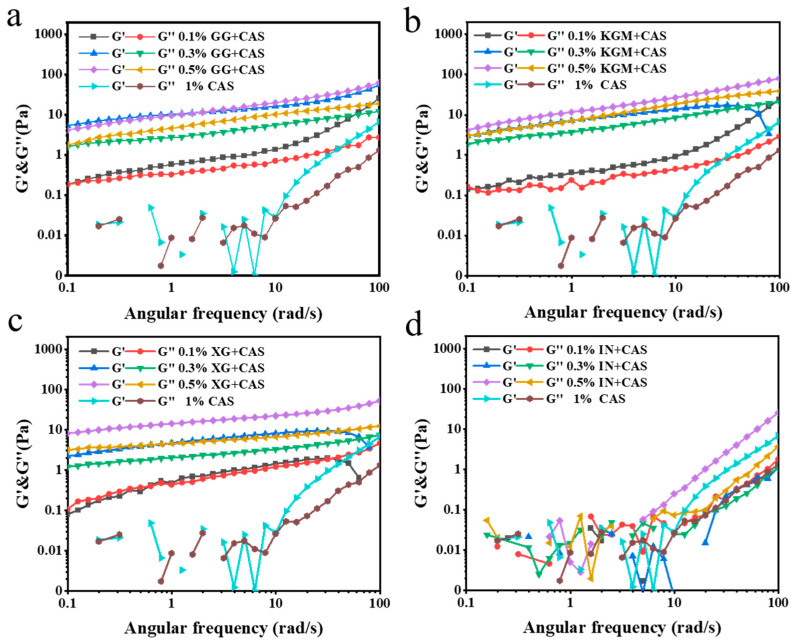
Frequency sweeps of GG/CAS emulsions (**a**), KGM/CAS emulsions (**b**), XG/CAS emulsions (**c**), and IN/CAS emulsions (**d**).

The dynamic rheological properties of emulsions are key parameters for assessing their ability to build network structures and the mechanical properties of the system. As shown in Figure 5, the energy storage modulus (G′) > loss modulus (G″) for all the samples indicates that the emulsion system tends to be in a solid-like state. Remarkably, the emulsion G′ shows a clear frequency dependence, indicating that the emulsions are still “liquid” to some extent. It suggested that the difference between emulsion and gel behavior may stem from the fluidity and plasticity of the emulsions under high-frequency scanning conditions [22]. This property further suggests the presence of a well-developed network structure in the emulsions. Among all polysaccharide-improved emulsions, XG showed the most significant enhancement, with significantly higher G′ and G″ than the other polysaccharides at high concentrations (0.3% and 0.5%). This was attributed to the extension of its rigid molecular chains and the electrostatic barrier effect to further enhance the spatial positional resistance between the droplets and the mechanical coupling of molecular chains, which constructs a highly dense and stable network structure with excellent elastic properties [23]. KGM and GG were closer to each other in terms of improvement. This indicates that both polysaccharides have similar mechanisms of action in emulsion systems and can build a network of certain strength through the physical entanglement of molecular chains. The uneven distribution of the branched chains of GG weakened the structural integrity of the network, resulting in a slightly lower strength than that of the KGM/CAS emulsions. The IN/CAS emulsions failed to maintain stable mechanical responses under fluid shear, with discontinuities in the measurement data, demonstrating that IN is ineffective in regulating the stability of the emulsions.

### 2.5. Oscillation Amplitude Sweeps of Polysaccharide/CAS Emulsions

**Figure 6:**
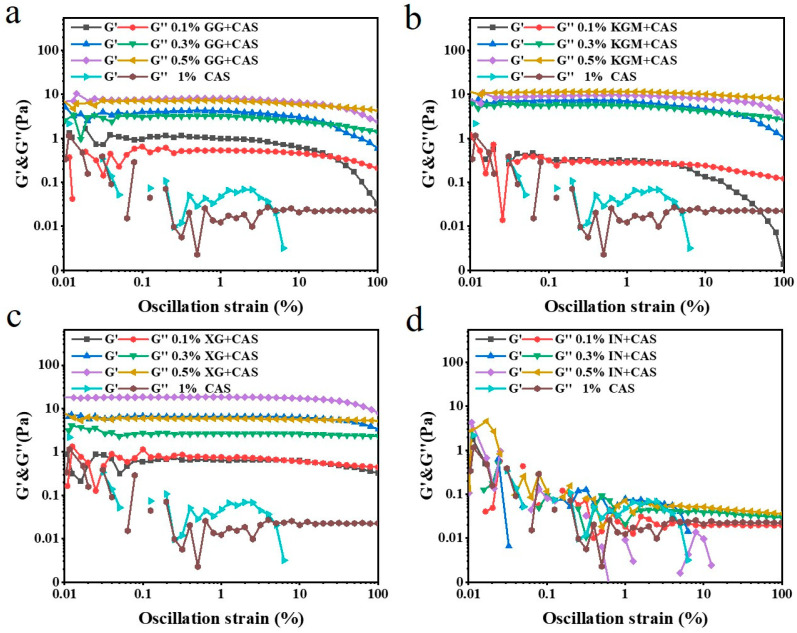
Oscillation amplitude of GG/CAS emulsions (**a**), KGM/CAS emulsions (**b**), XG/CAS emulsions (**c**), and IN/CAS emulsions (**d**).

Frequency sweeps are inadequate for practical exploration of emulsions, whereas oscillation amplitude sweeps reveal the regulation of polysaccharide characteristics on the network strength and strain tolerance of CAS emulsions. As illustrated in Figure 6, in the linear viscoelastic region (LVER), G′ and G″ remained stable for all samples. As the strain magnitude increased, the emulsions exhibited a typical “strain-thinning” phenomenon, which is in agreement with the results of Espert et al. [24]. It is proven that the network formation rate decreases while the loss rate increases under large strain conditions. The polysaccharide molecular chains undergo an oriented arrangement consistent with the flow field, weakening molecular chain entanglement and leading to partial disintegration of the overall network strength [25]. XG/CAS emulsions exhibited optimal strain tolerance with maximum resistance to large deformations. The critical strain point of its G′ was significantly lagged and decreased less. This indicates that its electrostatic barrier effect and intermolecular interactions maintained the integrity of the network and the interfacial stability between droplets under high strain [26]. Compared with KGM/CAS emulsions, GG/CAS emulsions had stronger strain-thinning behavior. Especially at low concentrations, the uneven distribution of branched chains hindered intermolecular entanglement and cross-linking, weakening the network strength and accelerating the rate of network disintegration under high strain conditions. IN, due to its low molecular weight and weak intermolecular interactions, exhibited the poorest strain resistance and failed to improve the emulsion properties.

### 2.6. Microstructural Observation

**Figure 7:**
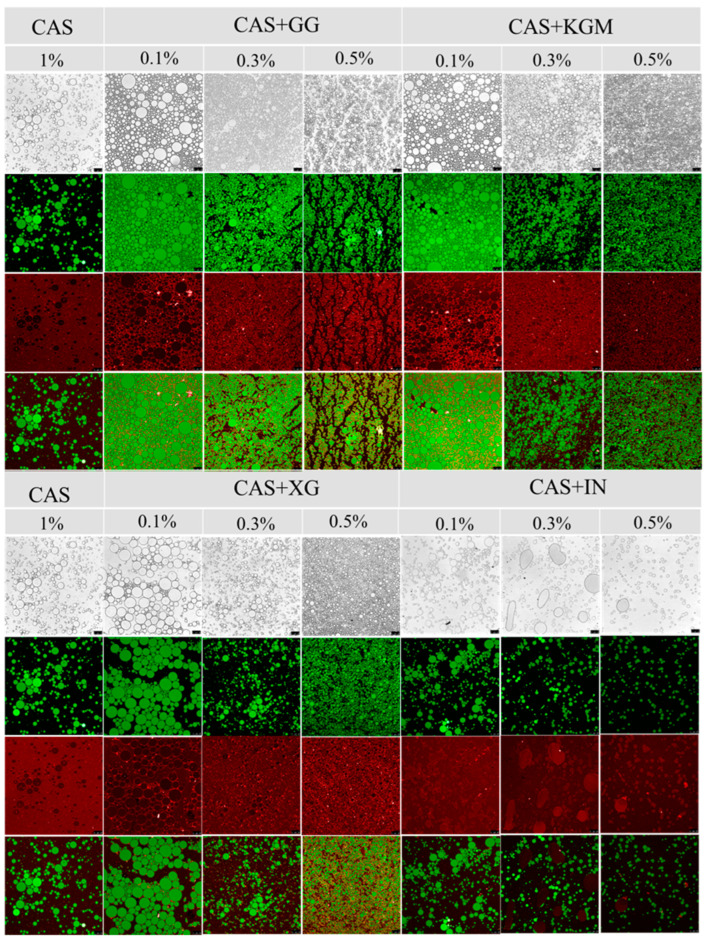
CLSM images of polysaccharide/CAS emulsions (green: oil phase, red: aqueous phase). Scale bar: 25 µm.

CLSM images (Figure 7) revealed that the emulsions formed a typical O/W emulsion structure. In the CAS emulsions, the droplet size was large and unevenly distributed, and there was obvious aggregation and fusion between droplets. The addition of polysaccharide significantly improved the droplet distribution and the integrity of the interfacial layer, and the droplet size decreased significantly with the increase of polysaccharide concentration and the distribution was more homogeneous. Especially in the high-concentration (0.5%) group, where the droplets showed a small and dispersed structure. Notably, XG showed optimal regulation at different concentrations with the most uniform emulsion distribution. These microscopic features were highly consistent with the previous results of rheological properties and particle size distribution, which further clarified the mechanism of polysaccharide action. The polysaccharide formed a dense or thick interfacial layer on the droplet surface through intermolecular interactions (Figures S1 and S2) together with CAS, which fundamentally enhanced the mechanical strength of the interface and effectively prevents droplet aggregation. Meanwhile, the polysaccharide formed a stable three-dimensional network in the aqueous phase through the electrostatic barrier, self-assembly effect (XG), and intermolecular entanglement (KGM and GG) [27,28], which increased fluid viscosity and limited droplet migration and coalescence, thereby enhancing emulsion stability.

**Figure 8:**
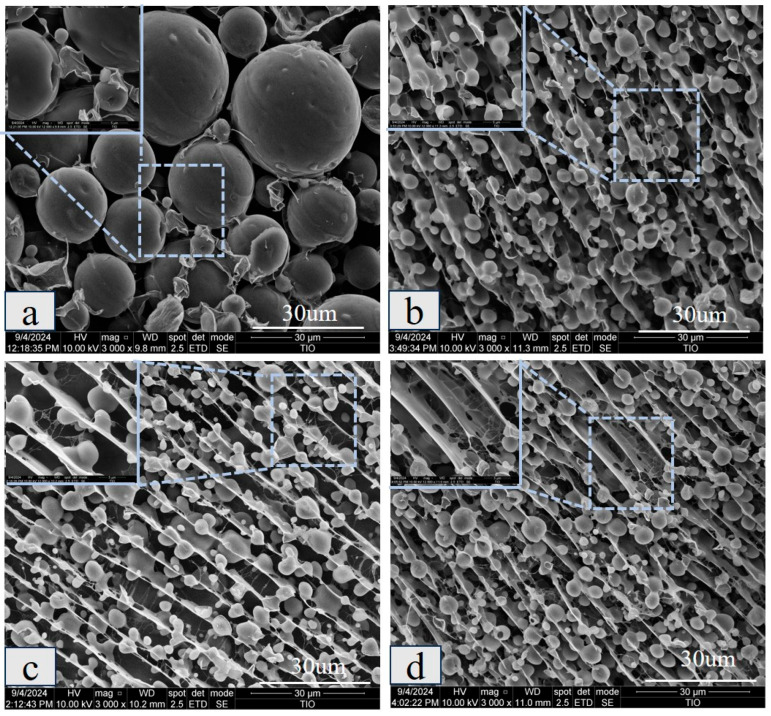
Cryo-SEM images of CAS emulsions (**a**), GG/CAS emulsions (**b**), KGM/CAS emulsions (**c**), and XG/CAS emulsions (**d**) at 0.5% polysaccharide concentration.

Figure 8 further reveals the interfacial layer properties of the emulsion and the network connections between the droplets. The results show that a few membrane connections were present on the surface of the CAS emulsion droplets, and the overall distribution was disordered, with individual droplets, indicating insufficient mechanical strength of the interfacial membrane. After the addition of polysaccharide, the droplet size decreased, and the membrane structure on the droplet surface became more obvious, with the network connection becoming tighter. Especially in KGM/CAS and XG/CAS emulsions, the polysaccharide chains were effectively connected with the droplet surface membrane, and a dense network structure was constructed between the droplets [29]. KGM enhanced the connectivity between the droplets through intermolecular entanglement, forming a continuous support structure [30], while XG formed a thick interfacial layer on the droplet surface by virtue of rigid molecular chains and the electrostatic barrier effect. Simultaneously, a highly regular microscopic network was constructed to further inhibit droplet migration and aggregation. In comparison, the GG/CAS emulsions had a looser membrane structure and network connections, with some gaps between droplets. These microscopic features provide direct evidence for the synergistic mechanism of polysaccharides in the interfacial reinforcement of emulsions and the construction of three-dimensional networks.

### 2.7. Antioxidant Activity of Loaded Curcumin Polysaccharide/CAS Emulsions

**Figure 9:**
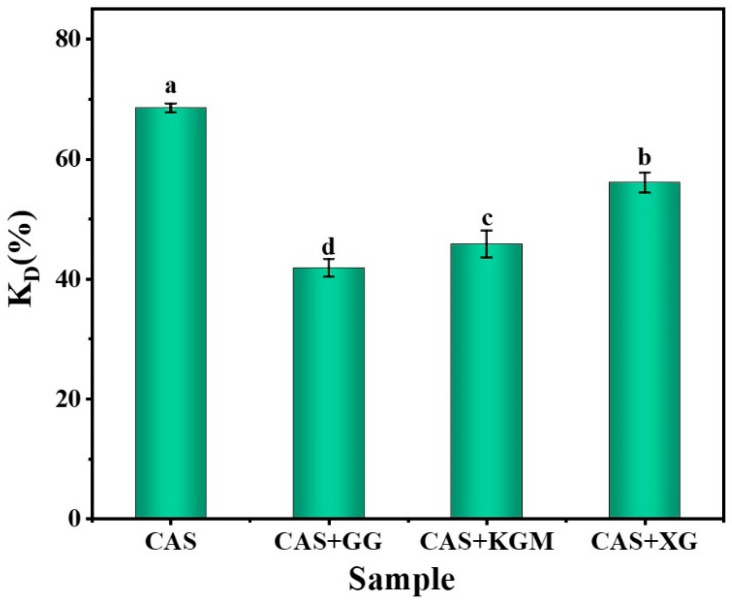
Antioxidant activity of polysaccharide/CAS emulsions (0.5% polysaccharide). Lowercase letters indicate significant differences in k_D_ values of different polysaccharide emulsions (*p* < 0.05).

Figure 9 measures the DPPH radical scavenging (K_D_; antioxidant capacity) of different emulsion systems loaded with curcumin. The results showed that CAS emulsions had the highest K_D_, while among the emulsions with added polysaccharides, XG/CAS emulsions exhibited the optimal antioxidant capacity. Instead, the high scavenging rate resulted from the instability of the emulsion, where droplet aggregation and rupture released large amounts of curcumin into the aqueous phase, allowing full interaction with DPPH [31]. However, the XG/CAS emulsion significantly enhanced emulsion stability (*p* < 0.05) by forming a thick and stable interfacial film on the droplet surface through a strong electrostatic barrier effect. This interfacial reinforcement reduced curcumin loss, while the highly regular microscopic network formed by polysaccharide chains increased emulsion viscosity, which limited free radical diffusion and migration. This allowed the released curcumin to capture DPPH radicals more efficiently. Additionally, the introduction of XG polyhydroxy side chains provided more protons, leading to enhanced free radical scavenging and improved antioxidant capacity [32]. The antioxidant property of the neutral polysaccharide KGM itself resulted in a slightly higher DPPH radical scavenging rate of its emulsion than that of the GG/CAS emulsion.

### 2.8. FFA Release Behavior of Polysaccharide/CAS Emulsion

**Figure 10:**
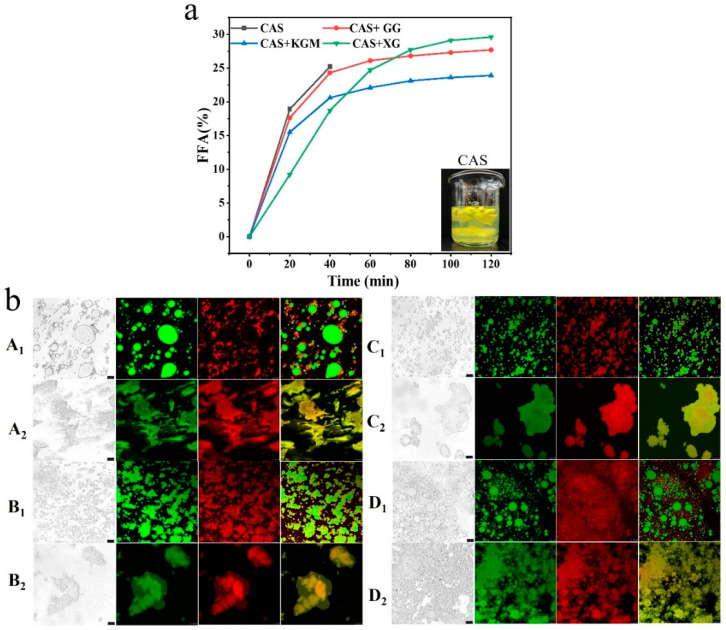
FFA release (**a**) and CLSM images (**b**) of CAS emulsion with 0.5% polysaccharide after gastrointestinal digestion (A: polysaccharide free, B: GG, C: KGM, D: XG, 1: gastric phase, and 2: intestine phase, green: oil phase, red: aqueous phase). Scale bar: 25 µm.

Figure 10a demonstrates FFA release from emulsions under simulated gastrointestinal digestion, assessing the capacity of different polysaccharides to regulate lipid digestion and create an optimal lipid environment. The results showed that the CAS emulsion was destabilized in the early stage of digestion, in which a large amount of FFA was rapidly released. The emulsions disintegrated at 40 min, showing a clear digestion-intolerant behavior. The addition of polysaccharides displayed superior digestion resistance, which significantly slowed down the rate of FFA release. The XG/CAS emulsions effectively inhibited FFA release in the early stages, attributable to the thick interfacial layer and electrostatic barrier on the droplet surface, which may have prevented enzymatic and bile-salt-induced droplet disruption. This facilitated slow lipid release, creating a stable lipid environment for curcumin dissolution and transport, thereby reducing degradation and potentially improving absorption efficiency [33]. Interestingly, the electrostatic barrier of XG/CAS emulsions significantly inhibited droplet disruption in the early stages of digestion. However, as digestion progressed, the electrostatic repulsion may have been neutralized by bile salts and enzymes, leading to droplet coalescence, increased lipid surface exposure, and accelerated lipid release [34]. This exhibited a characteristic of initial slow release followed by rapid release, making it suitable for phased curcumin delivery systems (enteric-targeted delivery systems) [35]. GG/CAS emulsions show a high FFA release rate at the beginning of digestion, which suggests limited interfacial protection and a poorly constructed stable environment for lipid solubilization. KGM/CAS emulsions exhibited a sustained and steady FFA release, with intermolecular entanglements forming a dense interfacial layer on the droplet surface. Simultaneously, a stable three-dimensional network structure was established in the aqueous phase, providing continuous mechanical support to the droplets. This property renders it suitable for long-lasting and stable curcumin delivery.

### 2.9. Microstructure of Emulsion During In Vitro Digestion

Figure 10b further visualizes the microstructure of the emulsion after gastrointestinal digestion. The results indicate that under the influence of pepsin and acidic conditions, emulsion droplets in the gastric phase underwent varying degrees of aggregation and deformation, with small droplets gradually merging into large ones. The interfacial membrane of the CAS emulsion degraded under the combined effects of gastric acid and pepsin hydrolysis, which resulted in the loss of droplet protection. Short-range attractive forces dominated, causing rapid droplet coalescence and the formation of large droplet aggregates [36]. In contrast, polysaccharide/CAS emulsions significantly enhanced resistance to acidity and enzymes, demonstrating stronger anti-coalescence properties, thereby effectively delaying the process of emulsion destabilization in the gastric phase. The XG/CAS emulsion showed the best performance due to the formation of a thick interfacial layer on the droplet surface. After entering the intestine phase, the interfacial protection ability and aqueous phase network structure of the emulsion system were gradually lost with the continuous action of bile salts and lipase. At the end of intestinal digestion, the structure of all emulsions had collapsed, and the droplets had completely lost their dispersibility, which was manifested by droplet coalescence and disordered release of lipid. This suggests that the stability of the emulsions was irreversibly damaged during prolonged enteric digestion.

## 3. Conclusions

In this study, a comparative study was conducted in the regulation of CAS emulsion properties by XG, KGM, GG, and IN. IN could not improve the CAS emulsion properties, while the molecular chains of XG, KGM, and GG significantly inhibited droplet aggregation and reduced droplet size through effective binding to the droplet surface film. The stability was significantly improved with the increase of the polysaccharide concentration. The addition of XG, KGM, and GG increased both G′ and G″ and slowed down the “strain-thinning” phenomenon of the emulsion under applied strain. The order of the differential effects of polysaccharide-regulated CAS emulsions was XG > KGM > GG > IN. The polysaccharide-regulated lipid release displayed different forms, creating a more stable lipid environment for curcumin dissolution and transport in the gastrointestinal tract. Among them, the XG/CAS emulsion showed excellent slow-release behavior, presenting a staged pattern. These findings provide rational options for improving the stability of emulsion systems and provide some theoretical support for the development of functional emulsions to meet different needs.

## 4. Materials and Methods

### 4.1. Materials

CAS (protein content ≥ 90%) was purchased from Sinopharm Group Chemical Reagent Co., Ltd. (Shanghai, China). KGM (MW 1.4 × 10^6^ Da) was supplied by Hubei Yizhi Konjac Industry Co., Ltd. (Hubei, China). XG and GG were purchased from Shanghai Yuanye Biotechnology Co., Ltd. (Shanghai, China). IN (DP ≥ 23) was purchased from Cosucra (Warcoing, Belgium). Camellia oil was purchased from Henan Ludashan Camellia Oil Co., Ltd. (Xinyang, China). Pepsin (≥1200 U/g), lipase (30,000 U/g), and other reagents were purchased from Sinopharm Chemical Reagent Co., Ltd. (Shanghai, China). The remaining reagents were of analytical grade.

### 4.2. Preparation of Polysaccharide/CAS Emulsions

CAS solution (1 wt%) was prepared by dissolving CAS powder in ultrapure water and stirring at 25 °C for 2 h. The solution was then hydrated at 4 °C and stored overnight. Different concentrations (0 wt%, 0.1 wt%, 0.3 wt%, and 0.5 wt%) of XG, KGM, GG, and IN were added to the CAS solution and stirred for 6 h at 25 °C to prepare the aqueous phases, and 20% camellia oil was used as the oil phase (curcumin concentration 0.1 wt%). The mixtures were homogenized using the rotor–stator homogenizer (diameter: 13 mm, IKA Works Co., Ltd., Guangzhou, China) for 3 min at 12,000 rpm to prepare the CAS emulsions, XG/CAS emulsions, KGM/CAS emulsions, GG/CAS emulsions, and IN/CAS emulsions (20 mL) at 25 °C. All the emulsions were allowed to stand at room temperature for 2 h to reach a stable state before further characterization.

### 4.3. Determination of Particle Size

Emulsion (1 mL) was diluted 1000 times to avoid multiple scattering effects. The emulsion sauter mean diameter (D_3,2_) was determined using a Malvern laser particle sizer (Master Sizer 3000, Malvern, UK; Formula (1)) [37]. Samples were equilibrated for 60 s before recording. The refractive index was 1.330 for water and 1.470 for camellia oil:

D_3,2_ = (Σn_i_D_i_^3^/Σn_i_D_i_^2^) × 100, (1)

where n_i_ is the number of droplets, and D_i_ is the diameter of the droplets.

### 4.4. Storage Stability Analysis

The prepared emulsions were transferred to glass bottles, and the emulsions were stored at 4 °C for 14 days. The change in emulsion creaming index (CI) was measured at 0, 1, 3, 5, 7, 9, 11, and 13 days, respectively. The CI of the emulsions was calculated according to Equation (2) [38]:

CI (%) = (H_s_/H_e_) × 100, (2)

where H_s_ and H_e_ are, respectively, the layered and total height of the emulsion (mm).

### 4.5. Rheological Measurements

Emulsions were theologically tested using a DHR-2 rheometer (TA Instruments, New Castle, DE, USA). All flow sweeps, frequency sweeps, and large-amplitude oscillation strain were performed on a 40 mm diameter aluminum plate with a 1 mm gap. For steady-state behavior, the shear rate was increased from 0.01 s^−1^ to 100 s^−1^ at 25 °C. Frequency sweeps were performed by increasing the frequency from 0.1 Hz to 100 Hz with a strain of 0.5%. Oscillation amplitude sweeps were performed by increasing the strain from 0.01% to 100% at a frequency of 1 Hz.

### 4.6. Microstructural Observations

The microforms of the emulsions were visualized using CLSM (SP8, Leica, Wetzlar, Germany) and Cryo-SEM (Quanta 450, FEI, Hillsboro, OR, USA). For CLSM, the microstructure of the stained samples was observed with a 40× objective. Nile Red and Nile Blue A were excited at 488 and 633 nm, respectively. For Cryo-SEM, the emulsion micro-morphology was observed under high vacuum at 10.0 kV.

### 4.7. Determination of Antioxidant Activity

The antioxidant activity was measured by the 2,2-diphenyl-1-picrylhydrazyl (DPPH) assay [39]. The emulsion was diluted 0.5 times by adding 0.2 mL to 4 mL of DPPH solution (0.198 mM) and incubated for 20 min at room temperature away from light. The absorbance was read at 517 nm using a UV-Vis spectrophotometer (Lambda 465, PerkinElmer, Waltham, MA, USA). DPPH (%) was calculated by the following equation:

DPPH (%) = [(Ab_s0_ − Ab_s1_)/Ab_s0_)] × 100, (3)

where Ab_s0_ is the absorbance of pure DPPH and Ab_s1_ is the absorbance of the emulsion after incubation with light.

### 4.8. In Vitro Digestion Analysis

A slight modification of the previous method was followed to simulate the gastrointestinal digestion behavior [40]. Gastric phase: 1 L of distilled water with 2 g NaCl, 7 mL HCl, and 3.2 g pepsin was used to adjust the pH to 2, with 1 M HCl to simulate gastric fluid. The emulsion sample was mixed with gastric fluid (1:1) and digested at 100 rpm at 37 °C for 2 h. Intestinal phase: lipase (4.8 mg/mL), bile salt solution (5 mg/mL), and CaCl_2_ (750 mM) were mixed to simulate intestinal fluid. The above digestive solution was mixed with intestinal fluid (1:1), the pH of the mixture was adjusted to 7, and it was incubated at 37 °C and 150 rpm for 2 h to simulate intestinal digestion. During this process, we used CLSM to observe the microstructure of the emulsion after digestion in the gastric and intestinal phases, respectively. Lipid digestion behavior was determined as the percentage of FFA released during digestion, and the amount of FFA released was judged by the amount of 1 M NaOH required to neutralize the released FFA:

FFA (%) = (VCM/2m) × 100,(4)

where V and C are the volume (L) and concentration (mol/L) of NaOH, respectively, while m and M are the initial mass (g) and relative molecular weight (g/mol) of the oil, respectively.

### 4.9. Statistical Analysis

All the experiments were conducted in triplicate. Graphs were plotted using Origin2021. Statistical differences (*p* < 0.05) were determined by analysis of variance (ANOVA) and Duncan’s test using SPSS 27.

## Data Availability

Research data are available from the authors.

## Supplementary Materials

The following supporting information can be downloaded at: https://www.mdpi.com/article/10.3390/gels11120968/s1. Figure S1. Fourier transform infrared spectroscopy spectrum of polysaccharide/CAS solution; Figure S2. Differential Scanning Calorimetry of polysaccharide/CAS solution.
