# Supplementary material for: Physical and Gastrointestinal Digestive Properties of Sodium Caseinate Emulsions Regulated by Four Different Polysaccharides
Source: Gels. 2025 Dec 1;11(12):968. doi: 10.3390/gels11120968 (PMC12732494; doi:10.3390/gels11120968)
Supplement: Supplementary file 1 [file gels-11-00968-s001.zip › gels-4007585-supplementary.pdf]

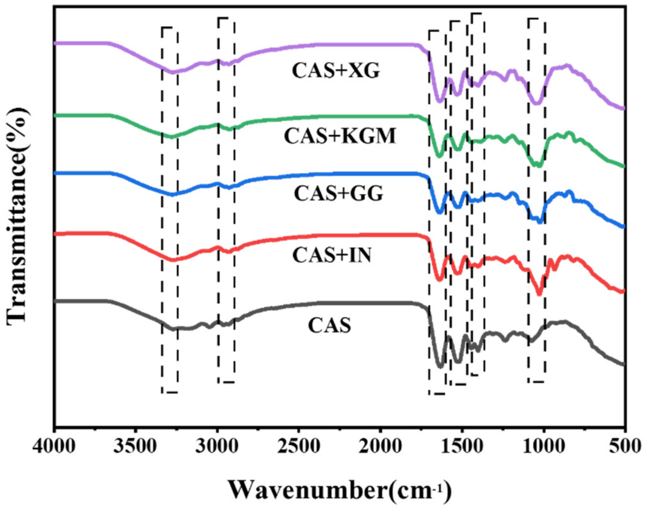

Figure S1. Fourier transform infrared spectroscopy spectrum of polysaccharide/CAS solution.

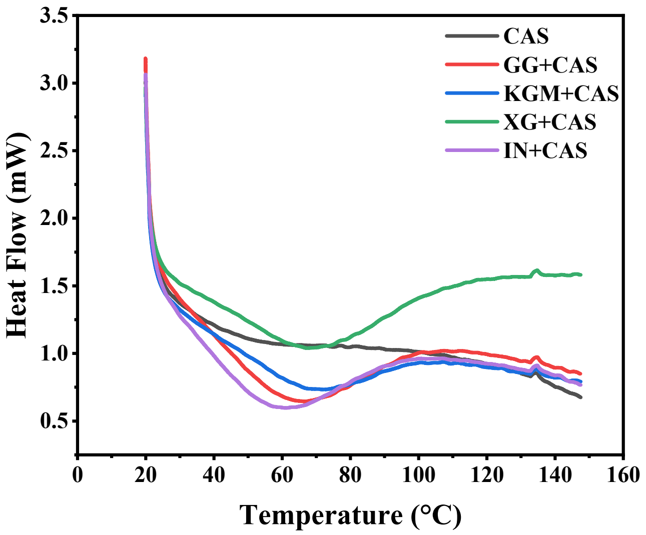

Figure S2. Differential Scanning Calorimetry of polysaccharide/CAS solution.
